# 2,6-Dimethyl-*N*-(2-methyl­phen­yl)-1,3-dioxan-4-amine

**DOI:** 10.1107/S1600536813025294

**Published:** 2013-09-18

**Authors:** Zeenat Fatima, Gottimukkala Rambabu, Bandapalli Palakshi Reddy, Vijayaparthasarathi Vijayakumar, Devadasan Velmurugan

**Affiliations:** a Centre of Advanced Study in Crystallography and Biophysics, University of Madras, Guindy Campus, Chennai 600 025, India; b Chemistry Department, GEBH, Sree Vidyanikethan Engineering College, A. Rangampet, Tirupati 517102, India; c Centre for Organic and Medicinal Chemistry, VIT University, Vellore 632 014, India

## Abstract

In the title compound, C_13_H_19_NO_2_, the dioxane ring adopts a chair conformation and its mean plane makes a dihedral angle of 45.36 (8)° with the phenyl ring. In the crystal, mol­ecules are linked by pairs of N—H⋯O hydrogen bonds, forming inversion dimers with *R* ^2^_2_(12) ring motifs. These dimers are consolidated by pairs of C—H⋯O hydrogen bonds with *R* ^2^_2_(8) ring motifs.

## Related literature

For applications of 1,3-dioxane derivatives, see: Wang *et al.* (1996*a* ▶,*b* ▶); Yuan *et al.* (2005 ▶). Dioxane rings are frequently encountered in many bioactive mol­ecules, some of which are cytotoxic agents (Aubele *et al.*, 2005 ▶) and anti­muscarinic agents (Marucci *et al.*, 2005 ▶). For related crystal structures, see: Chuprunov *et al.* (1981 ▶); Thevenet *et al.* (2010 ▶); Fatima *et al.* (2013 ▶). For hydrogen-bond motifs, see: Bernstein *et al.* (1995 ▶).
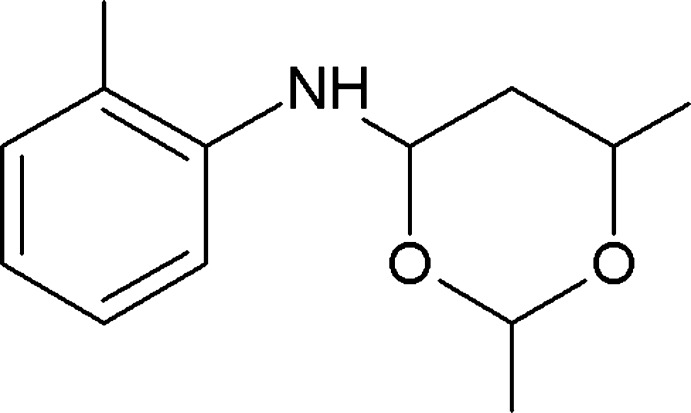


## Experimental

### 

#### Crystal data

C_13_H_19_NO_2_
*M* *_r_* = 221.29
Monoclinic, 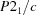

*a* = 8.0209 (2) Å
*b* = 7.8762 (2) Å
*c* = 20.4293 (5) Å
β = 99.066 (2)°
*V* = 1274.48 (6) Å^3^
*Z* = 4
Mo *K*α radiation
μ = 0.08 mm^−1^
*T* = 293 K
0.30 × 0.25 × 0.20 mm

#### Data collection

Bruker SMART APEXII area-detector diffractometer
Absorption correction: multi-scan (*SADABS*; Bruker, 2008 ▶) *T*_min_ = 0.692, *T*_max_ = 0.746
12359 measured reflections
3177 independent reflections
2481 reflections with *I* > 2σ(*I*)
*R*_int_ = 0.019

#### Refinement

*R*[*F* ^2^ > 2σ(*F* ^2^)] = 0.040
*wR*(*F* ^2^) = 0.119
*S* = 1.03
3177 reflections
152 parameters
H atoms treated by a mixture of independent and constrained refinement
Δρ_max_ = 0.18 e Å^−3^
Δρ_min_ = −0.12 e Å^−3^

### 

Data collection: *APEX2* (Bruker, 2008 ▶); cell refinement: *SAINT* (Bruker, 2008 ▶); data reduction: *SAINT*; program(s) used to solve structure: *SHELXS97* (Sheldrick, 2008 ▶); program(s) used to refine structure: *SHELXL97* (Sheldrick, 2008 ▶); molecular graphics: *ORTEP-3 for Windows* (Farrugia, 2012 ▶) and Macrae *et al.*, 2008 ▶); software used to prepare material for publication: *SHELXL97* and *PLATON* (Spek, 2009 ▶).

## Supplementary Material

Crystal structure: contains datablock(s) global, I. DOI: 10.1107/S1600536813025294/su2643sup1.cif

Structure factors: contains datablock(s) I. DOI: 10.1107/S1600536813025294/su2643Isup2.hkl

Supplementary material file. DOI: 10.1107/S1600536813025294/su2643Isup3.cml

Additional supplementary materials:  crystallographic information; 3D view; checkCIF report

## Figures and Tables

**Table 1:** Hydrogen-bond geometry (Å, °)

*D*—H⋯*A*	*D*—H	H⋯*A*	*D*⋯*A*	*D*—H⋯*A*
N1—H1⋯O2^i^	0.843 (15)	2.559 (15)	3.3688 (13)	161.3 (13)
C3—H3*A*⋯O1^i^	0.97	2.54	3.4950 (13)	167

Symmetry code: (i) 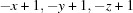.

**Fig. 1.**
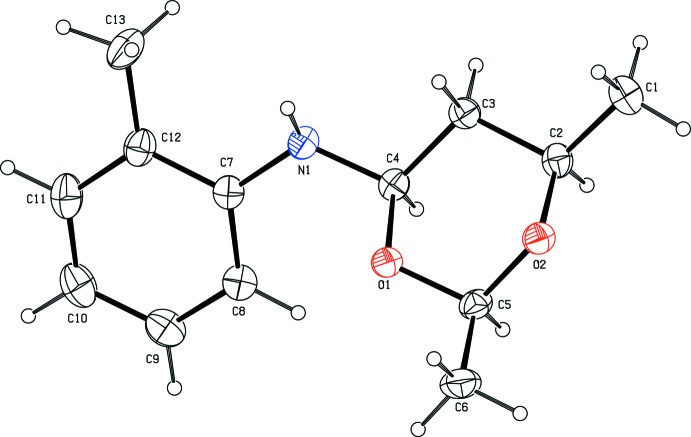
The molecular structure of the title molecule, with atom labelling. Displacement ellipsoids are drawn at the 30% probability level.

**Fig. 2.**
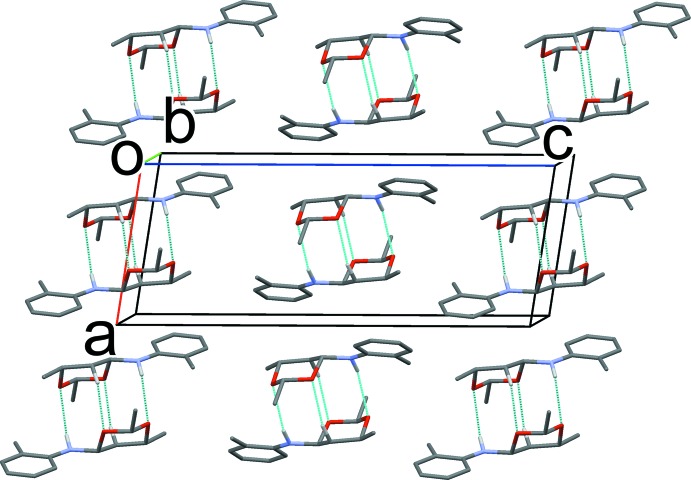
A view along the b axis of the crystal packing of the title compound. The N-H···O and C-H···O hydrogen bonds are shown as dashed lines (see Table 1 for details; H atoms not involved in hydrogen bonding have been omitted for clarity).

## supplementary crystallographic information

### 1. Comment

1,3-dioxane derivatives have applications in the pharmaceutical (Wang *et al.*, 1996*b*) and cosmetics industry (Wang *et al.*, 1996*a*; Yuan *et al.*, 2005). Dioxane rings are frequently encountered in many bioactive molecules, some of which are cytotoxic agents (Aubele *et al.*, 2005) and antimuscarinic agents (Marucci *et al.*, 2005). In view of the excellent biological and pharmacological applications of this class of compounds, we have undertaken the synthesis of the title compound and report herein on its crystal structure.

In the title molecule, Fig. 1, the dioxane ring (O1/O2/C2—C5) adopts a *chair* conformation and its mean plane makes a dihedral angle of 45.36 (8)° with the phenyl ring (C7—C12).

In the crystal, molecules are linked by a pair of N-H···O hydrogen bonds forming inversion dimers with an *R*^2^_2_(12) ring motif (Bernstein *et al.*, 1995). These dimers are consolidated by a pair of C-H···O hydrogen bonds with an *R*^2^_2_(8) ring motif (Table 1 and Fig. 2).

### 2. Experimental

To 2-toulidine (1 mmol), acetaldehyde (3 mmol) was added drop wise and the mixture was stirred for ca. 4 h at 273 K. The progress of the reaction was monitored through TLC. On completion the reaction the mixture was washed with petroleum ether. The resultant mixture was dissolved in diethylether and the solvent allowed to evaporate. The solid product obtained was recrystallized with diethylether to yield block-like colourless crystals, suitable for X-ray diffraction analysis.

### 3. Refinement

The NH H atom was located in a difference Fourier map and freely refined. The C bound H atoms were placed in calculated positions and refined as riding atoms: C—H = 0.93 - 0.98 Å with *U*_iso_(H) = 1.5*U*_eq_(C-methyl) and = 1.2*U*_eq_(C) for other H atoms.

### Crystal data

**Table:**

C_13_H_19_NO_2_	*F*(000) = 480
*M**_r_* = 221.29	*D*_x_ = 1.153 Mg m^−^^3^
Monoclinic, *P*2_1_/*c*	Mo *K*α radiation, λ = 0.71073 Å
Hall symbol: -P 2ybc	Cell parameters from 3177 reflections
*a* = 8.0209 (2) Å	θ = 2.0–28.4°
*b* = 7.8762 (2) Å	µ = 0.08 mm^−^^1^
*c* = 20.4293 (5) Å	*T* = 293 K
β = 99.066 (2)°	Block, colourless
*V* = 1274.48 (6) Å^3^	0.30 × 0.25 × 0.20 mm
*Z* = 4	

### Data collection

**Table:**

Bruker SMART APEXII area-detector diffractometer	3177 independent reflections
Radiation source: fine-focus sealed tube	2481 reflections with *I* > 2σ(*I*)
Graphite monochromator	*R*_int_ = 0.019
ω and φ scans	θ_max_ = 28.4°, θ_min_ = 2.0°
Absorption correction: multi-scan (*SADABS*; Bruker, 2008)	*h* = −10→10
*T*_min_ = 0.692, *T*_max_ = 0.746	*k* = −10→10
12359 measured reflections	*l* = −26→27

### Refinement

**Table:**

Refinement on *F*^2^	Secondary atom site location: difference Fourier map
Least-squares matrix: full	Hydrogen site location: inferred from neighbouring sites
*R*[*F*^2^ > 2σ(*F*^2^)] = 0.040	H atoms treated by a mixture of independent and constrained refinement
*wR*(*F*^2^) = 0.119	*w* = 1/[σ^2^(*F*_o_^2^) + (0.0553*P*)^2^ + 0.1687*P*] where *P* = (*F*_o_^2^ + 2*F*_c_^2^)/3
*S* = 1.03	(Δ/σ)_max_ < 0.001
3177 reflections	Δρ_max_ = 0.18 e Å^−^^3^
152 parameters	Δρ_min_ = −0.12 e Å^−^^3^
0 restraints	Extinction correction: *SHELXL*, Fc^*^=kFc[1+0.001xFc^2^λ^3^/sin(2θ)]^-1/4^
Primary atom site location: structure-invariant direct methods	Extinction coefficient: 0.077 (4)

### Special details

**Table:**

Geometry. All e.s.d.'s (except the e.s.d. in the dihedral angle between two l.s. planes) are estimated using the full covariance matrix. The cell e.s.d.'s are taken into account individually in the estimation of e.s.d.'s in distances, angles and torsion angles; correlations between e.s.d.'s in cell parameters are only used when they are defined by crystal symmetry. An approximate (isotropic) treatment of cell e.s.d.'s is used for estimating e.s.d.'s involving l.s. planes.
Refinement. Refinement of *F*^2^ against ALL reflections. The weighted *R*-factor *wR* and goodness of fit *S* are based on *F*^2^, conventional *R*-factors *R* are based on *F*, with *F* set to zero for negative *F*^2^. The threshold expression of *F*^2^ > σ(*F*^2^) is used only for calculating *R*-factors(gt) *etc*. and is not relevant to the choice of reflections for refinement. *R*-factors based on *F*^2^ are statistically about twice as large as those based on *F*, and *R*- factors based on ALL data will be even larger.

### Fractional atomic coordinates and isotropic or equivalent isotropic displacement parameters (Å^2^)

**Table:**

	*x*	*y*	*z*	*U*_iso_*/*U*_eq_	
H1	0.3087 (18)	0.4830 (19)	0.5783 (7)	0.067 (4)*	
C1	0.31391 (18)	0.68745 (18)	0.35171 (7)	0.0682 (4)	
H1A	0.3217	0.6610	0.3064	0.102*	
H1B	0.4212	0.7278	0.3737	0.102*	
H1C	0.2300	0.7738	0.3531	0.102*	
C2	0.26506 (14)	0.53043 (15)	0.38614 (6)	0.0509 (3)	
H2	0.1570	0.4884	0.3627	0.061*	
C3	0.24940 (13)	0.56014 (14)	0.45810 (5)	0.0474 (3)	
H3A	0.3511	0.6147	0.4804	0.057*	
H3B	0.1550	0.6354	0.4607	0.057*	
C4	0.22287 (12)	0.39494 (14)	0.49256 (5)	0.0457 (3)	
H4	0.1121	0.3489	0.4739	0.055*	
C5	0.35637 (15)	0.25112 (14)	0.41290 (6)	0.0511 (3)	
H5	0.2477	0.2066	0.3910	0.061*	
C6	0.4939 (2)	0.12583 (18)	0.40646 (8)	0.0727 (4)	
H6A	0.4992	0.1072	0.3604	0.109*	
H6B	0.4703	0.0204	0.4267	0.109*	
H6C	0.6000	0.1697	0.4281	0.109*	
C7	0.19069 (13)	0.28783 (15)	0.60341 (5)	0.0475 (3)	
C8	0.13383 (15)	0.12993 (17)	0.57909 (7)	0.0570 (3)	
H8	0.1240	0.1079	0.5339	0.068*	
C9	0.09169 (17)	0.00538 (19)	0.62110 (8)	0.0684 (4)	
H9	0.0524	−0.0991	0.6040	0.082*	
C10	0.10746 (19)	0.0348 (2)	0.68794 (8)	0.0773 (4)	
H10	0.0799	−0.0494	0.7163	0.093*	
C11	0.16463 (19)	0.1905 (2)	0.71237 (7)	0.0739 (4)	
H11	0.1760	0.2096	0.7578	0.089*	
C12	0.20590 (14)	0.31981 (18)	0.67183 (6)	0.0573 (3)	
C13	0.2628 (2)	0.4902 (2)	0.69976 (7)	0.0778 (4)	
H13A	0.2537	0.4935	0.7460	0.117*	
H13B	0.1929	0.5772	0.6768	0.117*	
H13C	0.3781	0.5088	0.6943	0.117*	
N1	0.22865 (13)	0.41904 (13)	0.56182 (5)	0.0508 (2)	
O1	0.35151 (9)	0.27540 (10)	0.48107 (4)	0.0477 (2)	
O2	0.39299 (10)	0.40450 (10)	0.38245 (4)	0.0509 (2)	

### Atomic displacement parameters (Å^2^)

**Table:**

	*U*^11^	*U*^22^	*U*^33^	*U*^12^	*U*^13^	*U*^23^
C1	0.0724 (8)	0.0649 (8)	0.0671 (8)	0.0025 (7)	0.0106 (6)	0.0160 (6)
C2	0.0435 (5)	0.0577 (7)	0.0495 (6)	−0.0018 (5)	0.0009 (4)	0.0032 (5)
C3	0.0407 (5)	0.0495 (6)	0.0513 (6)	0.0054 (4)	0.0047 (4)	−0.0003 (5)
C4	0.0375 (5)	0.0529 (6)	0.0461 (6)	−0.0011 (4)	0.0045 (4)	−0.0026 (4)
C5	0.0567 (6)	0.0469 (6)	0.0500 (6)	−0.0108 (5)	0.0095 (5)	−0.0089 (5)
C6	0.0930 (10)	0.0516 (7)	0.0778 (9)	0.0049 (7)	0.0270 (8)	−0.0136 (6)
C7	0.0377 (5)	0.0567 (7)	0.0486 (6)	0.0031 (4)	0.0085 (4)	0.0021 (5)
C8	0.0502 (6)	0.0623 (7)	0.0587 (7)	−0.0030 (5)	0.0096 (5)	0.0005 (6)
C9	0.0576 (7)	0.0630 (8)	0.0861 (10)	−0.0058 (6)	0.0158 (6)	0.0088 (7)
C10	0.0706 (9)	0.0835 (11)	0.0808 (10)	−0.0003 (8)	0.0211 (7)	0.0279 (8)
C11	0.0714 (8)	0.0987 (12)	0.0537 (7)	0.0022 (8)	0.0161 (6)	0.0137 (7)
C12	0.0497 (6)	0.0738 (8)	0.0497 (6)	0.0029 (6)	0.0122 (5)	−0.0002 (6)
C13	0.0895 (10)	0.0930 (11)	0.0544 (8)	−0.0102 (9)	0.0217 (7)	−0.0172 (7)
N1	0.0514 (5)	0.0556 (6)	0.0455 (5)	−0.0050 (4)	0.0079 (4)	−0.0035 (4)
O1	0.0496 (4)	0.0458 (4)	0.0475 (4)	0.0004 (3)	0.0073 (3)	−0.0025 (3)
O2	0.0523 (4)	0.0504 (5)	0.0517 (4)	−0.0042 (3)	0.0136 (3)	−0.0022 (3)

### Geometric parameters (Å, º)

**Table:**

C1—C2	1.5048 (17)	C6—H6B	0.9600
C1—H1A	0.9600	C6—H6C	0.9600
C1—H1B	0.9600	C7—C8	1.3893 (17)
C1—H1C	0.9600	C7—N1	1.4014 (15)
C2—O2	1.4376 (14)	C7—C12	1.4065 (16)
C2—C3	1.5133 (16)	C8—C9	1.3800 (18)
C2—H2	0.9800	C8—H8	0.9300
C3—C4	1.5103 (16)	C9—C10	1.371 (2)
C3—H3A	0.9700	C9—H9	0.9300
C3—H3B	0.9700	C10—C11	1.375 (2)
C4—N1	1.4212 (14)	C10—H10	0.9300
C4—O1	1.4431 (13)	C11—C12	1.386 (2)
C4—H4	0.9800	C11—H11	0.9300
C5—O2	1.4108 (14)	C12—C13	1.501 (2)
C5—O1	1.4122 (13)	C13—H13A	0.9600
C5—C6	1.5010 (18)	C13—H13B	0.9600
C5—H5	0.9800	C13—H13C	0.9600
C6—H6A	0.9600	N1—H1	0.843 (15)
			
C2—C1—H1A	109.5	C5—C6—H6C	109.5
C2—C1—H1B	109.5	H6A—C6—H6C	109.5
H1A—C1—H1B	109.5	H6B—C6—H6C	109.5
C2—C1—H1C	109.5	C8—C7—N1	122.26 (10)
H1A—C1—H1C	109.5	C8—C7—C12	119.20 (11)
H1B—C1—H1C	109.5	N1—C7—C12	118.51 (11)
O2—C2—C1	107.58 (9)	C9—C8—C7	120.85 (12)
O2—C2—C3	109.08 (8)	C9—C8—H8	119.6
C1—C2—C3	113.24 (10)	C7—C8—H8	119.6
O2—C2—H2	109.0	C10—C9—C8	120.41 (14)
C1—C2—H2	109.0	C10—C9—H9	119.8
C3—C2—H2	109.0	C8—C9—H9	119.8
C4—C3—C2	111.04 (9)	C9—C10—C11	119.03 (14)
C4—C3—H3A	109.4	C9—C10—H10	120.5
C2—C3—H3A	109.4	C11—C10—H10	120.5
C4—C3—H3B	109.4	C10—C11—C12	122.41 (14)
C2—C3—H3B	109.4	C10—C11—H11	118.8
H3A—C3—H3B	108.0	C12—C11—H11	118.8
N1—C4—O1	109.70 (8)	C11—C12—C7	118.09 (13)
N1—C4—C3	111.35 (9)	C11—C12—C13	121.14 (12)
O1—C4—C3	109.23 (8)	C7—C12—C13	120.76 (12)
N1—C4—H4	108.8	C12—C13—H13A	109.5
O1—C4—H4	108.8	C12—C13—H13B	109.5
C3—C4—H4	108.8	H13A—C13—H13B	109.5
O2—C5—O1	111.04 (9)	C12—C13—H13C	109.5
O2—C5—C6	108.50 (10)	H13A—C13—H13C	109.5
O1—C5—C6	108.08 (10)	H13B—C13—H13C	109.5
O2—C5—H5	109.7	C7—N1—C4	121.93 (10)
O1—C5—H5	109.7	C7—N1—H1	115.0 (10)
C6—C5—H5	109.7	C4—N1—H1	112.4 (10)
C5—C6—H6A	109.5	C5—O1—C4	112.35 (8)
C5—C6—H6B	109.5	C5—O2—C2	111.57 (8)
H6A—C6—H6B	109.5		
			
O2—C2—C3—C4	−52.82 (11)	N1—C7—C12—C13	−0.45 (17)
C1—C2—C3—C4	−172.57 (9)	C8—C7—N1—C4	−4.57 (16)
C2—C3—C4—N1	172.86 (8)	C12—C7—N1—C4	177.47 (10)
C2—C3—C4—O1	51.55 (11)	O1—C4—N1—C7	−65.48 (12)
N1—C7—C8—C9	−177.63 (11)	C3—C4—N1—C7	173.48 (9)
C12—C7—C8—C9	0.31 (17)	O2—C5—O1—C4	60.92 (11)
C7—C8—C9—C10	−0.9 (2)	C6—C5—O1—C4	179.82 (9)
C8—C9—C10—C11	0.5 (2)	N1—C4—O1—C5	−177.70 (9)
C9—C10—C11—C12	0.5 (2)	C3—C4—O1—C5	−55.39 (11)
C10—C11—C12—C7	−1.0 (2)	O1—C5—O2—C2	−61.96 (11)
C10—C11—C12—C13	178.05 (14)	C6—C5—O2—C2	179.40 (9)
C8—C7—C12—C11	0.63 (17)	C1—C2—O2—C5	−179.23 (9)
N1—C7—C12—C11	178.65 (11)	C3—C2—O2—C5	57.58 (11)
C8—C7—C12—C13	−178.47 (12)		

### Hydrogen-bond geometry (Å, º)

**Table:**

*D*—H···*A*	*D*—H	H···*A*	*D*···*A*	*D*—H···*A*
N1—H1···O2^i^	0.843 (15)	2.559 (15)	3.3688 (13)	161.3 (13)
C3—H3*A*···O1^i^	0.97	2.54	3.4950 (13)	167

Symmetry code: (i) −*x*+1, −*y*+1, −*z*+1.
